# Predicting Factors Influencing Visual Function of the Eye in Patients with Unresolved Facial Nerve Palsy after Upper Eyelid Gold Weight Loading

**DOI:** 10.3390/jcm10040578

**Published:** 2021-02-04

**Authors:** Izabela Nowak-Gospodarowicz, Marek Rękas

**Affiliations:** Department of Ophthalmology, Military Institute of Medicine, 04-141 Warsaw, Poland; mrekas@wim.mil.pl

**Keywords:** facial nerve palsy, exposure keratopathy, lagophthalmos, gold weights, lid loading

## Abstract

Implantation of gold weights into the upper eyelid is a proven method of treating lagophthalmos and exposure keratopathy in patients with unresolved facial nerve palsy. The aim of this study was to evaluate the factors affecting visual acuity and corneal complications in patients after upper eyelid gold weight lid loading. Material and methods: This prospective consecutive clinical study was conducted in years 2012–2018. In total, 59 people (40 women, 19 men aged 55.5 ± 17.4 years) meeting the inclusion criteria were treated with gold weights. The ordered multinomial logit model was used to analyze the factors affecting best-corrected visual acuity (BCVA) and degree of exposure keratopathy after surgery. The influence of the following variables was analyzed: patient age, etiology and duration of the facial nerve palsy, history of the previous eyelid surgery, degree of lagophthalmos in mm, presence of Bell’s phenomenon, and corneal sensation, Schirmer test results. Results: Implantation of gold weights into the upper eyelid effectively reduced lagophthalmos and exposure keratopathy in the study group (*p* < 0.001). BCVA was maintained or better in 95% of patients after surgery. Patient age, presence of the Bell’s phenomenon, and corneal sensation significantly affected the final BCVA (*p* < 0.1). The presence of Bell’s phenomenon and corneal sensation had a positive effect on the degree of keratopathy after surgery (*p* < 0.1). In turn, patient age and history of tarsorrhaphy were significant negative prognostic factors of exposure keratopathy and BCVA after surgery (*p* < 0.05). Etiology and duration of facial nerve palsy, degree of corneal exposure in mm, and results of the Schirmer test did not have a significant impact on the outcome after surgery (*p* > 0.1). Conclusions: The results of our study may help to answer the question of how to direct ophthalmologists and other specialists who refer to ophthalmologists for management advice in patients with facial nerve palsy. Elderly patients with a history of tarsorrhaphy who present with poor Bell’s phenomenon and/or a lack of corneal sensation should be the first candidates for immediate correction of lagophthalmos.

## 1. Introduction

Facial nerve palsy (seventh cranial nerve) affects people all over the world, regardless of gender and race [1,2]. As a result of impairment of function of the facial nerve, closing the eye becomes impaired which may deteriorate the visual function of the eye, among other features [3,4,5]. This is the result of corneal damage, which, without protection from the eyelids, is constantly exposed to drying out and external factors [5,6,7,8]. Initial changes include corneal epithelial defects followed by infection, deep ulceration, corneal perforation and, as a consequence, loss of the eye [4,5,6,7]. There is a lack of standards for the treatment of paralytic lagophthalmos [7,8,9].

In some patients, ocular pain and pathologic corneal lesions are observed very quickly, within days of illness onset despite topical treatment with moisturizers and eye patching [4,7]. Other patients, even after a few years of illness, have good visual acuity and corneal changes are observed only after applying special methods of staining the eye surface by an ophthalmologist [2,10].

In light of contemporary scientific research and from a clinical point of view, it is important to identify risk factors for deterioration of visual acuity and corneal damage, which could give guidance for diagnostic and therapeutic management dedicated to this group of patients in the long run.

Therefore the aim of this study was to identify the factors affecting visual acuity and degree of exposure keratopathy in patients with unresolved facial nerve palsy who were given gold eyelid weights due to corneal complications of lagophthalmos.

## 2. Materials and Methods

### 2.1. Design of the Study

This prospective single center clinical consecutive study was conducted from 2012 to 2018 in accordance with the principles of good medical practice and after obtaining approval (ethical approval number 57/WIM/2011, received on 17 August 2011) from the Bioethical Commission at the Military Institute of Medicine in Warsaw (Warsaw, Poland). All patients provided written informed consent to participate in this study.

### 2.2. Criteria for Participation in the Study Included

(1) Unresolved facial palsy and unchanged lagophthalmos for at least 3 months despite intensive rehabilitation, (2) ocular symptoms reported by the patient due to exposure keratopathy not responding to conservative treatment (topical treatment with moisturizers and eye patching or moist chamber), (3) at least good function (>4 mm) of the levator muscle of the upper eyelid, and (4) condition of both the skin of the eyelid and ocular muscle of the eye allowed for surgery.

Patients were excluded (1) if they were younger than 18 years, (2) were unable or disagreed to participate in this study, and (3) did not attend follow-up visits.

### 2.3. The Characteristic of the Study Group

The study group included 59 patients: 40 women (67.8%) and 19 men (32.2%) with an average age of 55.5 ± 17.4 years. In 37 patients (62.7%), persistent palsy affected the left facial nerve, while in 22 (37.2%) the palsy was right-sided. Etiologies of the facial nerve palsy in the study group were as follows: cerebellopontine angle tumor surgery in 46 (78%) patients, salivary gland tumor surgery in 5 (8.5%), trauma in 4 (6.8%), congenital facial nerve palsy in 2 (3.4%), and idiopathic unresolved facial nerve palsy in 2 (3.4%). All patients presented with at least grade 4 disfunction of the facial nerve according to the House Brackman scale [11]. The average Sunnybrook score was 28.6 ± 12.2 [12]. Patients used an average of 9 ± 6 drops of moisturizing drugs per day. The mean intraocular pressure was 15 ± 3 mmHg.

### 2.4. Clinical Assessment

Best corrected visual acuity (BCVA) was measured on the Snellen chart prior to surgery and at each follow-up visit.

Degree of lagophthalmos was measured in mm by caliper when attempting eyelid closure. The marginal reflex index 1 (MRD1, the distance between the upper eyelid margin and corneal reflex) and function of the levator muscle of the upper eyelid (LF) was measured with a caliper in mm. Where LF was more than 10 mm was assessed as very good and good where was 4–10 mm. Patients with weak levator function (<4 mm) were not included in this study.

Ocular surface disorders were classified based on slit lamp examination and corneal fluoresceine staining as grade 1–4, where 1 = no pathological changes, 2 = epithelial defects (epitheliopathy), 3 = stromal lesions, and 4 = deep corneal ulcer or corneal perforation [13].

Corneal sensation and blink reflex was assessed as: normal, weakened, or absent with the use of a cotton swab by one person (IN-G), where normal = evident reaction with blinking to touching with a swab, weakened = patient reported that the touch was felt, but reaction might not be evident, and absent = no reaction and no perception reported to the touch.

Bell’s phenomenon was assessed as very good (the eye rolls upward on attempted closure of the eyelids, there is no corneal exposure), incomplete (upward eye movement is present, but there is partial corneal exposure), and absent (no eye movement during attempted eyelid closure).

The Schirmer test was performed after applying anesthetic drops and was considered abnormal below 10 mm [14,15].

Upper eyelid gold weight loading involved high pretarsal fixation with levator recession as previously described [16] and was performed under an operating microscope in every patient.

Correction of paralytic ectropion was performed concomitantly, if necessary. Depending on clinical condition, medial spindle, lateral tarsal strip, or combination of both techniques was performed [17,18,19].

Follow-ups for evaluation of objective results of treatment, cosmetic effect of the surgery and complications were done on the following time schedule: day 1, 10, month 1, 3, 6, 12, 24, 36, and once a year thereafter. All patients from the study group remain under the care of our outpatient clinic.

### 2.5. Statistical Analysis

Statistical analysis was performed using SPSS software (IBM Corp. Released 2012. IBM SPSS Statistics for Windows, Version 21.0. Armonk, NY: IBM Corp, USA). For measurable features, the normality of the distribution of analyzed parameters was evaluated using the Shapiro–Wilk test. The Wilcoxon pair order test was used to compare the two dependent groups. For the two independent groups, the Mann–Whitney U test was used. To assess the relationship between BCVA and degree of keratopathy with selected features a multinomial logit model was built. The influence of the following 8 variables was analyzed: patient age, etiology and duration of the facial nerve palsy, history of previous eyelid surgery, degree of lagophthalmos in mm, presence of Bell’s phenomenon, corneal sensation, and Schirmer test result.

In the multinomial logit model BCVA scores measured on the Snellen chart were divided into 4 groups according to distribution quartile: 0 to <0.25 (first distribution quartile), 0.25 to <0.5 (second distribution quartile), 0.5 to <0.75 (third distribution quartile), and 0.75–1.0 (fourth distribution quartile).

For the multinomial logit model, ocular surface disorders were classified based on the slit lamp examination and corneal fluoresceine staining as grade 1–4, where 1 = no pathological changes, 2 = epithelial defects (epitheliopathy), 3 = stromal lesions, and 4 = deep corneal ulcer or corneal perforation [13]. Because only 1 patient from the study group presented with a keratopathy grade 4 after surgery, in the logit model the relationships for keratopathy grades 3 and 4 were examined together.

Patient age, duration of facial nerve palsy and degree of lagophthalmos in mm were considered as continuous variables. Presence of Bell’s phenomenon, presence of corneal sensation, etiology of the facial nerve palsy (cerebellopontine angle tumor vs. other), history of tarsorrhaphy, and Schirmer test result were considered as binary variables.

In the logit model a significance level of *p* < 0.1 was assumed to indicate the existence of statistically significant differences or dependencies while for the others analysis a significance level of *p* < 0.05 was considered significant.

## 3. Results

Sixty four patients with corneal complications from lagophthalmos due to unresolved facial nerve palsy were treated with upper eyelid gold weight loading in our ward from 2012 to 2018. Data from five patients enrolled in the study were not included because the patient either passed away (2) or did not attend follow-up visits (3); therefore, data from 59 patients were analyzed.

The average duration of facial nerve palsy was 116 ± 202 months.

Twenty one patients (35.6%) had no surgical history of treatment prior to study enrollment. In 21 patients (35.6%) tarsorrhaphy was performed one or more times to allow corneal healing, of which 5 (8.5%) patients were additionally treated with an amniotic membrane transplant to the cornea elsewhere. In eight patients (13.5%), facial nerve/sublingual nerve anastomosis was previously applied with no satisfactory results. In seven patients (11.8%) correction of the paralytic ectropion of the lower eyelid was performed elsewhere. Two other patients (3.4%) had a history of upper eyelid gold weight loading, which was then removed elsewhere due to complications.

Function of the levator muscle of the upper eyelid, measured with a caliper, was assessed as good in 33 patients (55.9%) and very good in 26 (44%).

The MRD1 averaged 3.9 ± 1.6 mm in the affected eye and 3.8 ± 0.9 mm in the contralateral eye.

The Schirmer test was considered abnormal in 12 cases (20.3%).

Lagophthalmos was on average 7 ± 3 mm prior to surgery.

The average weight of the implant was 1.5 ± 0.3 g.

Depending on clinical condition, correction of the paralytic ectropion was performed simultaneously with upper eyelid gold weight loading in 31 out of 59 patients (61%).

**Figure 1:**
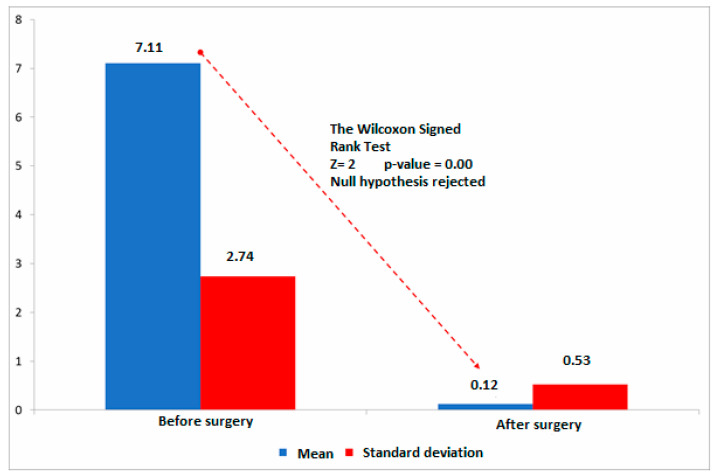
Changes in lagophthalmos (in mm) 6 months after upper eyelid gold weight loading. The Wilcoxon signed rank-test with Z as test statistic (the rank mean of one group compared to the overall rank mean).

Lagophthalmos was corrected in all patients after surgery. The postoperative changes are illustrated in Figure 1.

BCVA was on average 0.4 ± 0.3 prior to surgery.

**Figure 2:**
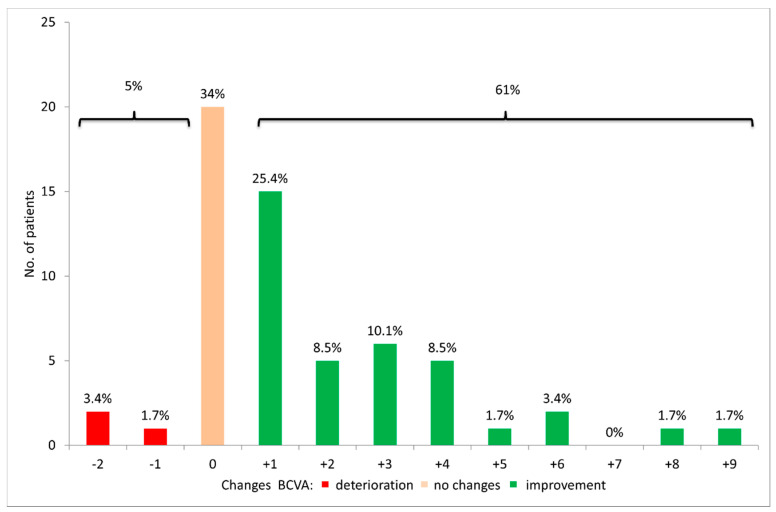
Best-corrected visual acuity (BCVA) changes 6 months after upper eyelid gold weight loading.

Improvement in BCVA after surgery was noted in 36 patients (61%), no changes in BCVA in 20 (33.9%), and deterioration in 3 (5%) (Figure 2).

BCVA stabilized in the study group approximately six months after surgery and did not change significantly in later follow-ups (*p* > 0.1).

**Figure 3:**
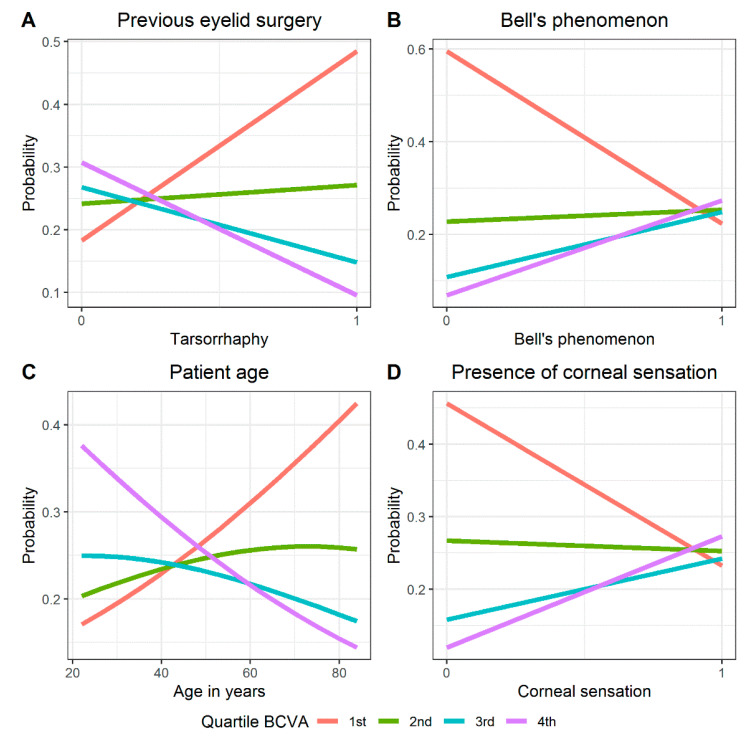
Results of the multinomial ordered logit model for postoperative BCVA with independent variable: (**A**)—previous eyelid surgery, (**B**)—Bell’s phenomenon, (**C**)—patient age, (**D**)—presence of corneal sensation, assuming 4 graded levels

In the logit model, a significant influence on postoperative BCVA was demonstrated at a level of *p* < 0.1 for the following variables: patient age, previous eyelid surgery (tarsorrhaphy), Bell’s phenomenon and corneal sensation (Table 1; Figure 3A–D). A positive regression coefficient value indicates that Bell’s phenomenon and corneal sensation are variables that directly contribute to the transition of BCVA from worse to better. Values of the OR (odds ratio) indicate that patients with very good or even incomplete Bell’s phenomenon had a more than 18 times greater chance of better postoperative BCVA than patients in whom this phenomenon did not occur (Table 1; Figure 3B).

Similarly, preserved corneal sensation was associated with a 13-fold increase in chances for better BCVA after surgery compared to patients with no corneal sensation (Figure 3D; Table 1). A negative regression coefficient value indicates age and previous interventions (tarsorrhaphy) are transition factors from better to worse BCVA. With age, the chances for low BCVA (first distribution quartile) increased and decreased for a high postoperative BCVA (fourth distribution quartile).

In the study group, the cut-off point was estimated to be about 48 years of age (Figure 3C). History of tarsorrhaphy was associated with an increased chance of low postoperative BCVA (first quartile distribution) (Figure 3A).

Bell’s phenomenon was very good in 23 patients (39%), incomplete in 25 cases (42.4%), and absent in 11 patients (18.6%).

The categorization of Bell’s phenomenon (very good, present, incomplete and none) differentiates the study group in terms of achieved postoperative BCVA (*p* = 0.011). The differentiation is due to a significant difference in postoperative BCVA in groups with very good and no Bell’s phenomenon (*p* = 0.037) (Table 2).

Exposure keratopathy was found in all patients preoperatively. Epitheliopathy was present in 31 patients (52.5%). Pathology within the deeper layers of the cornea (i.e., interstitial changes) was noted in 14 patients (23.7%). The remaining 14 patients (23.7%) presented with deep corneal ulcers threatening perforation.

**Figure 4:**
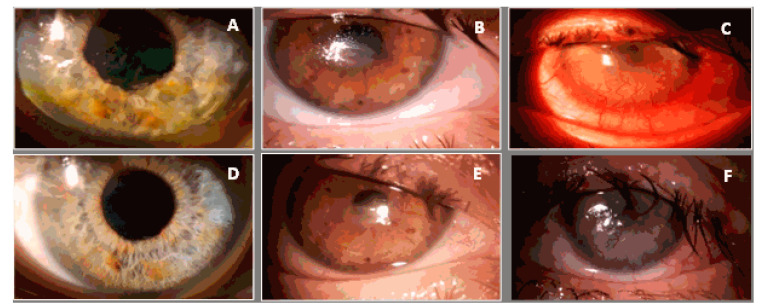
Pictures from a biomicroscope showing improvement of exposure keratopathy after surgery (on top: (**A**–**C**): pictures before surgery, on bottom: (**D**–**F**): the same corneas 6 months after surgery).

Clinical improvement of the surface of the eye was observed in all patients within 6 months after surgery (*p* < 0.001) (Table 3, Figure 4).

**Figure 5:**
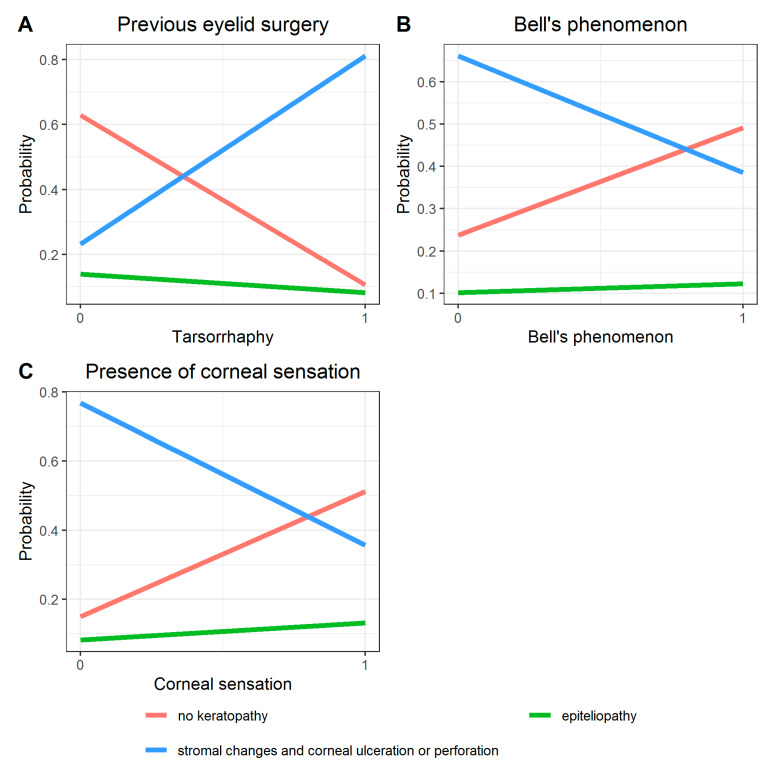
Results of multinomial ordered logit model for keratopathy after surgery with independent variable: (**A**)—previous eyelid surgery, (**B**)—Bell’s phenomenon, (**C**)—presence of corneal sensation, receiving 3 graded levels

In the logit model, a significant influence on the degree of keratopathy after surgery was demonstrated at a level of *p* < 0.1 for three variables: previous eyelid surgery (tarsorrhaphy), Bell’s phenomenon, and corneal sensation (Table 4; Figure 5A–C).

Tarsorrhaphy was performed in 21 patients (35.6%) prior to the upper eyelid gold weight loading procedure. There was permanent lateral tarsorrhaphy in eight patients, temporary lateral tarsorrhaphy in six and temporary complete tarsorrhaphy in seven patients.

The OR value indicates that patients who previously had undergone tarsorrhaphy had a greater than 20 times lower chance of healing the cornea after surgery compared to patients who had no previous eyelid surgery (Table 4; Figure 5A). The presence of Bell’s phenomenon and corneal sensation was associated with a greater chance of complete resolution of keratopathy after surgery (Table 4; Figure 5B,C). The impact of other variables turned out to be statistically insignificant (Table 4).

## 4. Discussion

Visual acuity is the basic parameter of an ophthalmological examination enabling quick assessment of the condition of the eye [6]. The correct visual acuity measured on standardized charts indicates the integrity of the structures of the anterior and posterior segment of the eye, visual pathway and visual cortex. The human cornea, which is physiologically a transparent structure, devoid of blood vessels, in addition to ensuring the continuity of the eyeball, allows the penetration of visual stimuli into the eye and focuses stimuli on the retina, which determines proper vision [1,3,6].

Keratopathy is a major cause of visual acuity deterioration and the main reason to seek ophthalmologic advice in patients with facial nerve palsy and lagophthalmos [5,6,7,8,20].

Exposure keratopathy was diagnosed in all patients enrolled in the study group preoperatively (Table 3).

Clinical improvement of the eye surface was observed in all patients within six months after surgery (*p* < 0.001) (Table 3; Figure 4). The best results were obtained in patients with epitheliopathy at baseline—in this group total resolution of exposure keratopathy was observed in 84% patients. This was reflected in BCVA improvement, which was significantly improved in 61% of patients and maintained in another 34% of patients (Figure 2).

On the other hand, in the case of deep corneal stromal changes and severe ulcerations observed in almost half of patients before surgery, eye surface condition improved, but in most of these patients no significant improvement or even deterioration of BCVA was noted due to the post-inflammatory scars of the cornea located in the visual axis (Figure 4; Table 3) [21].

There are several mechanisms responsible for protecting the cornea. Among them, clinically important mechanisms include the correct positioning and functioning of the eyelids, the right amount and quality of the tear film, preserved corneal sensation and a good Bell’s phenomenon [1,6,9]. The eyelids protect the surface of the eye from drying out during sleep and provide cleansing and protection against the ingress of foreign bodies during the day through the blink reflex. By spreading the tear film on the surface of the eye and supporting the draining of tears into the tear points, eyelids ensure optimal hydration of the eye. Bell’s phenomenon is an additional defensive reflex that occurs during attempted eyelid closure and causes the eye to roll upwards hiding the cornea under the eyelids [1,7,8,20]. In turn, preserved sensation of the cornea is proven to be necessary to start repair mechanisms of the corneal epithelium [3,22,23].

MacIntosh, et al. [20] conclude that the official American Academy of Ophthalmology position on management of facial palsy should be updated in order to adequately direct ophthalmologists and other specialists who refer to ophthalmologists for management advice.

This is why, in our study, analysis of clinical data was performed on factors adversely affecting visual acuity and degree of corneal damage, which appear to be crucial from an ophthalmological point of view.

To investigate the impact variables including patient age, etiology and duration of facial nerve palsy, degree of lagophthalmos in mm, presence of Bell’s phenomenon, presence of corneal sensation and Schirmer’s test results a multinomial logit model was built. Analysis showed that only four variables (patient age, history of previous eyelid surgery (tarsorrhaphy), corneal sensation and Bell’s phenomenon) had a significant impact on postoperative BCVA (Table 1; Figure 3).

Patient age negatively affected postoperative BCVA in the study group (Table 1; Figure 3A). However, it should be noted that with age visual acuity deteriorates regardless of the presence of exposure keratopathy. In most cases this is caused by the aging process of the structures of the eye which most often are manifested in the form of cataracts, glaucoma or age-related macular degeneration (AMD) [24].

This finding was consistent with the insignificant *p*-value for the relationship between patient age and the degree of exposure keratopathy revealed in our study (Table 4).

Tarsorrhaphy is considered to be an alternative method of treatment to upper eyelid gold weight loading [1,6,25].

Although it is easy to perform, it may cause a limitation in visual field, scarring of eyelid tissues, trichiasis, and non-satisfactory cosmesis [1,20]. In our study a history of tarsorrhaphy was associated with worse BCVA and a more advanced degree of keratopathy after surgery (Table 1; Figure 3D). Patients who previously had tarsorrhaphy had a greater than 20 times lower chance for complete resolution of the exposure keratopathy after upper eyelid gold weight loading procedure compared to patients who had no history of previous eyelid surgery (Table 4; Figure 5A). Permanent tarsorrhaphy should not be recommended any more for patients who will require eyelid reanimation, but rather is recommended for comatose patients with poor overall prognosis who require protection of the globe [1,20].

Presence of Bell’s phenomenon and corneal sensation, which determines correct blink reflex were associated with better BCVA and a greater chance of complete resolution of exposure keratopathy after surgery compared to the condition of patients in whom Bell’s phenomenon and corneal sensation were absent (Table 1 and Table 4; Figure 5B,C). Patients with no Bell’s phenomenon had about two times worse postoperative BCVA than those whose Bell’s phenomenon was good or even incomplete (Table 2).

These findings are consistent with the results of a study conducted by Malthora et al., who in 2016, published the CADS protocol to assess ophthalmic involvement in patients with facial nerve palsy [9]. These authors highlight that the absence of Bell’s phenomenon, reduced corneal sensation and a Schirmer test of ≤5 mm should indicate a priority to provide cornea protection before treatment of asymmetry and synkinesis in patients with facial nerve palsy.

However, contrary to their findings, results of the Schirmer test as a factor influencing the BCVA and degree of exposure keratopathy were insignificant in our study (Table 1 and Table 4). At issue may be the questionable reliability and repeatability of the Schirmer test and its heterogenous reference values [9,14,15].

Pereira et al. conclude that decisions about the most appropriate method for treatment of paralytic lagophthalmos depend on the location, extent, degree and duration of paralysis, etiology, patient age, health, and expectations [8].

The general condition of the patient and their expectations are very important factors in planning the therapeutic procedure; however, degree of lagophthalmos, etiology and duration of facial palsy turned out to be statistically insignificant in our model (Table 1 and Table 4). More important than the measure of the degree of lagophthalmos (in mm) seems to be assessment of Bell’s phenomenon (which determines corneal exposure during attempted eyelid closure) and MRD 1 and MRD 2 to assess the eyelid position and asymmetry (which is necessary to plan an appropriate therapeutic procedure) [6,9,20].

Lee and Lew highlight in their study that prognosis and ophthalmic signs were worse when palsy of the third, fifth, or sixth cranial nerve was involved than in cases of isolated facial nerve palsy [26].

Because of their location, tumors and/or surgery of the cerebellopontine angle may cause damage to neighboring nerves in addition to the facial nerve [1,2,3]. This was the cause of facial nerve palsy in 78% of the patients from our study group. While it is obvious that ophthalmic signs may be worse in cases of concomitant multi cranial nerve injuries, the etiology of facial nerve palsy was not a significant variable directly influencing postoperative BCVA nor degree of keratopathy after surgery in our model (Table 1 and Table 4). The only significant variable was a lack of corneal sensation, which is a typical symptom of trigeminal nerve injury (ophthalmic branch of the fifth cranial nerve, nervus ophthalmicus), which strongly affects regeneration of the cornea [3,21,22,23] and the protective blink reflex [27] (Table 4, Figure 5C).

In light of previous studies [4,5,6,7,8,9,20,26], which stress that timely ophthalmologic examination and management of patients with facial nerve palsy are crucial to save their eyes, the results of our work were surprising, because the impact of duration of palsy were not statistically significant. (Table 1 and Table 4). The average duration of facial nerve palsy in the study group was 116 ± 202 months. Our results suggest that even this long facial nerve palsy duration had no significant influence on postoperative BCVA and degree of keratopathy. Given this, physicians should seek to answer the question, What does optimal time mean? The results of our study showed that this appropriate time depends on many factors, but complete resolution of exposure keratopathy after upper eyelid gold weight lid loading was possible only before the involvement of stromal changes of the cornea. Furthermore, the results of our study may answer the question of why some patients, despite a high degree of lagophthalmos and long-present facial nerve palsy, do not develop exposure keratopathy while others very quickly come to an ophthalmologist with severe ocular symptoms of lagophthalmos.

Results may also affect decision making in clinical practice given that not the duration of palsy, but above all a lack of Bell’s phenomenon and/or corneal sensation in combination with a previous history of tarsorrhaphy and/or advanced patient age are the main prognostic factors that should determine more intensive and more invasive treatment.

The corneal section of the CADS protocol aims to record the degree of exposure keratopathy and risk of sight-threatening decompensation based upon horizontal corneal exposure zones [9]. This evaluation is possible only by slit lamp examination which can be performed by an ophthalmologist.

Taking into account the results of the 23-year observational study of Joseph et al., who conclude that the majority of their patients did not require special ophthalmological treatment [10]; Lee and Lew [26]; and the results of our study, we advocate simplifying the basic assessment of eye condition in manifested lagophthalmos due to facial nerve palsy. In the CADS protocol [9], specifically the corneal section, we recommend an examination of Bell’s phenomenon and corneal sensation rather than a Schirmer test or measures of the corneal exposure zones as the basic assessment of eye condition. This examination is easy to perform by any physician and may determine an immediate referral to an ophthalmologist. Ophthalmologists, with specialized equipment, should be responsible for detailed examination of the eye zone. As advocated by MacIntosh et al., examination should include visual acuity, the brow, upper eyelid, lower eyelid, ocular surface and dynamic blinking [20,27]. This would help to provide more adequate treatment and seems to be economically justified.

It must be highlighted that our study has some limitations. The only technique used in the study group was the upper eyelid gold weight loading with levator recession and correction of paralytic ectropion. This method was selected based on clinical presentation, surgical experience and availability of materials in the selected medical center with good outcomes [28].

A statistic model was used to measure only selected variables with selected features with a significance value of <0.1 to <0.01. Nevertheless, to our knowledge this is the first analysis based not only on clinical experience but also on mathematical calculations that suggest future directions in clinical practice.

Thus, we believe that the results of our study may help to answer the still unresolved question of how to direct ophthalmologists and other specialists who refer to ophthalmologists for management advice in patients with facial nerve palsy [20].

## 5. Conclusions

The results of this study show that the best postoperative visual acuity after upper eyelid gold weight loading will be achieved in young patients with good Bell’s phenomenon and preserved corneal sensation. The assessment of Bell’s phenomenon and corneal sensation is easy to perform by any physician and may determine the need for an immediate referral for detailed ophthalmologic evaluation in patients with lagophthalmos due to facial nerve palsy. Timely repair of lagophthalmos (i.e., before stromal changes of the cornea) can lead to complete resolution of exposure keratopathy after upper eyelid gold weight lid loading.

We believe that the results of our study may help to answer the still unresolved question of how to direct ophthalmologists and other specialists who refer to ophthalmologists for management advice in patients with facial nerve palsy [20]. According to our study elderly patients with a history of tarsorrhaphy, poor Bell’s phenomenon, and/or a lack of corneal sensation should be the first candidates for immediate correction of lagophthalmos in order to save the visual function of their eyes.

## Figures and Tables

**Table 1:** Results of a multinomial ordered logit model examining the postoperative BCVA relationship with selected features.

Explantory Variables	Dependent Variable-Postoperative BCVA			
	Regression Coefficient	OR (Odds Ratio)	*p*-Value	Statistical Significance
Patient age	−0.025	1.011	0.032	**
History of previous eyelid surgery—tarsorrhaphy	−1.629	0.658	0.010	*
Etiology of the facial nerve palsy	0.824	9.477	0.245	NS
Duration of the facial nerve palsy	0.000	1.004	0.813	NS
Degree of lagophthalmos in mm	0.080	1.335	0.446	NS
Presence of Bell’s phenomenon	1.102	18.283	0.088	*
Schirmer test result	0.756	8.300	0.258	NS
Presence of corneal sensation	1.059	13.241	0.054	*
**Logit model diagnostics**				
AIC	163.371			
McFadden R^2^	0.544			
Log Likelihood	−70.685			

NS *p*-value > 0.1, * *p*-value ≤ 0.1, ** *p*-value ≤ 0.05.

**Table 2:** Relationship of postoperative BCVA with Bell’s phenomenon.

Bell’s Phenomenon	BCVA		Number of Observations	Mann–Whitney U Test		
	Mean	Standard Deviation		W	*p*-Value	Rejection of H_0_ *
Very good	0.62	0.36	23	183	0.037	Yes
None	0.35	0.2	11			
Very good	0.62	0.36	23	320.5	0.668	No
Incomplete	0.59	0.31	26			
None	0.35	0.2	11	83	0.045	Yes
Incomplete	0.59	0.31	26			

* H_0_ = null hypothesis = there is no difference between distributions of values in 2 compared groups.

**Table 3:** Changes in the degree of keratopathy in patients with facial nerve palsy after upper eyelid gold weight loading.

Patients with Facial Nerve Palsy	Keratopathy Grade (1–4)			
	1-No Pathological Changes	2-Epitheliopathy	3-Stromal Corneal Changes	4-Deep Corneal Ulcer or Corneal Perforation
Preoperative	0	31 (52.5%)	14 (23.7%)	14 (23.7%)
6 month follow-up	26 (44%)	7 (11.9%)	25 (42.4%)	1 (1.7%)
*p*-value (Wilcoxon rank test)	<0.001	<0.001	<0.001	<0.001

**Table 4:** Results of a multinomial logit model examining the postsurgical keratopathy grade relationship with selected features.

Explantory Variables	Dependent Variable-Keratopathy Grade			
	Regression Coefficient	OR (Odds Ratio)	*p*-Value	Statistical Significance
Patient’s age	0.031	1.031	0.264	NS
History of previous eyelid surgery—tarsorrhaphy	3.043	20.958	0.000	***
Etiology of the facial nerve palsy	−0.087	0.916	0.929	NS
Duration of the facial nerve palsy	0.002	1.002	0.362	NS
Degree of lagophthalmos in mm	−0.054	0.948	0.710	NS
Presence of Bell’s phenomenon	−1.735	0.176	0.091	*
Schirmer test result	−0.752	0.472	0.398	NS
Presence of corneal sensation	−2.114	0.121	0.035	**
**Logit model diagnostics**				
AIC	98.337			
McFadden R^2^	0.469			
Log Likelihood	−39.169			

NS *p*-value > 0.1, * *p*-value ≤ 0.1, ** *p*-value ≤ 0.05, *** *p*-value ≤ 0.01.
